# Secondary hyperparathyroidism, weight loss, and longer term mortality in haemodialysis patients: results from the DOPPS

**DOI:** 10.1002/jcsm.12722

**Published:** 2021-06-01

**Authors:** Hirotaka Komaba, Junhui Zhao, Suguru Yamamoto, Takanobu Nomura, Douglas S. Fuller, Keith P. McCullough, Pieter Evenepoel, Anders Christensson, Xinju Zhao, Mona Alrukhaimi, Fadwa Al‐Ali, Eric W. Young, Bruce M. Robinson, Masafumi Fukagawa

**Affiliations:** ^1^ Division of Nephrology, Endocrinology and Metabolism Tokai University School of Medicine Isehara Japan; ^2^ The Institute of Medical Sciences Tokai University Isehara Japan; ^3^ Arbor Research Collaborative for Health Ann Arbor MI USA; ^4^ Division of Clinical Nephrology and Rheumatology Niigata University Graduate School of Medical and Dental Sciences Niigata Japan; ^5^ Medical Affairs Department Kyowa Kirin Co., Ltd. Tokyo Japan; ^6^ Department of Nephrology and Renal Transplantation University Hospitals Leuven Leuven Belgium; ^7^ Laboratory of Nephrology, Department of Microbiology and Immunology KU Leuven Leuven Belgium; ^8^ Department of Nephrology, Skåne University Hospital Lund University Malmö Sweden; ^9^ Department of Nephrology Peking University People's Hospital Beijing China; ^10^ Department of Medicine Dubai Medical College Dubai United Arab Emirates; ^11^ Fahad Bin Jassim Kidney Center, Department of Nephrology Hamad General Hospital, Hamad Medical Corporation Doha Qatar

**Keywords:** Haemodialysis, Mortality, Secondary hyperparathyroidism, Weight loss

## Abstract

**Background:**

Wasting is a common complication of kidney failure that leads to weight loss and poor outcomes. Recent experimental data identified parathyroid hormone (PTH) as a driver of adipose tissue browning and wasting, but little is known about the relations among secondary hyperparathyroidism, weight loss, and risk of mortality in dialysis patients.

**Methods:**

We included 42,319 chronic in‐centre haemodialysis patients from the Dialysis Outcomes and Practice Patterns Study phases 2–6 (2002–2018). Linear mixed models were used to estimate the association between baseline PTH and percent weight change over 12 months, adjusting for country, demographics, comorbidities, and labs. Accelerated failure time models were used to assess 12 month weight loss as a mediator between baseline high PTH and mortality after 12 months.

**Results:**

Baseline PTH was inversely associated with 12 month weight change: 12 month weight loss >5% was observed in 21%, 18%, 18%, 17%, 15%, and 14% of patients for PTH ≥600 pg/mL, 450–600, 300–450, 150–300, 50–150, and <50 pg/mL, respectively. In adjusted analyses, 12 month weight change compared with PTH 150–299 pg/mL was −0.60%, −0.12%, −0.10%, +0.15%, and +0.35% for PTH ≥600, 450–600, 300–450, 50–150, and <50 pg/mL, respectively. This relationship was robust regardless of recent hospitalization and was more pronounced in persons with preserved appetite. During follow‐up after the 12 month weight measure [median, 1.0 (interquartile range, 0.6–1.7) years; 6125 deaths], patients with baseline PTH ≥600 pg/mL had 11% [95% confidence interval (CI), 9–13%] shorter lifespan, and 18% (95% CI, 14–23%) of this effect was mediated through weight loss ≥2.5%.

**Conclusions:**

Secondary hyperparathyroidism may be a novel mechanism of wasting, corroborating experimental data, and, among chronic dialysis patients, this pathway may be a mediator between elevated PTH levels and mortality. Future research should determine whether PTH‐lowering therapy can limit weight loss and improve longer term dialysis outcomes.

## Introduction

Wasting is a syndrome characterized by loss of fat and skeletal muscle that accompanies many chronic diseases including kidney failure.^1^, ^2^, ^3^ Observational studies have shown strong associations between signs of wasting, such as weight loss, and mortality risk in patients undergoing haemodialysis.^4^, ^5^ Individuals with kidney failure may consume less food due to dietary restrictions or loss of appetite,^6^, ^7^, ^8^, ^9^ but wasting is quite different from malnutrition caused by insufficient food intake.^2^, ^3^ One key characteristic of wasting is elevated basal energy expenditure, which leads to loss of adipose tissue and skeletal muscle through enhanced fat and protein catabolism.^2^, ^3^ Patients with kidney failure have been shown to have increased resting energy expenditure particularly in the presence of comorbidities.^10^, ^11^, ^12^

While the aetiology of wasting in kidney failure may be multifactorial,^2^, ^3^ recent experimental studies have demonstrated that parathyroid hormone (PTH) induces a phenotypic switch from white to brown adipocytes (a phenomenon termed adipose tissue browning) and thereby drives thermogenesis and hypermetabolism.^13^, ^14^ Secondary hyperparathyroidism (SHPT) is a common complication in haemodialysis patients.^15^, ^16^, ^17^ However, few studies have examined the potential link between SHPT and wasting in haemodialysis patients.^18^, ^19^, ^20^ Furthermore, SHPT has been shown to be associated with increased risk of death,^16^, ^17^ but the causal effect of SHPT on mortality is uncertain and underlying mechanisms not well understood.

We analysed data from the Dialysis Outcomes and Practice Patterns Study (DOPPS) to test the hypotheses that (i) SHPT leads to weight loss in haemodialysis patients and (ii) this pathway in part mediates the association of SHPT with mortality.

## Materials and methods

### Patients and data collection

The DOPPS (www.dopps.org) is an international prospective cohort study of patients 18 years or older treated with in‐centre haemodialysis. Details on study design and methods have been published.^21^, ^22^ Briefly, the study population was composed of randomly selected patients from a random sample of dialysis facilities within each country. Data for demographics, comorbid conditions, laboratory values, and prescriptions were abstracted from medical records using uniform and standardized data collection tools. Lack of appetite^23^ was collected in patient questionnaire (‘During the past four weeks, to what extent were you bothered by lack of appetite?’) at baseline in Phases 2–3, and annually in Phases 4–6. Mortality and hospitalization events were collected during study follow‐up. Study approval was obtained by a central institutional review board within each DOPPS country. Additional study approvals and patient consent were obtained as required by national and local ethics committee regulations.

**Figure 1:**
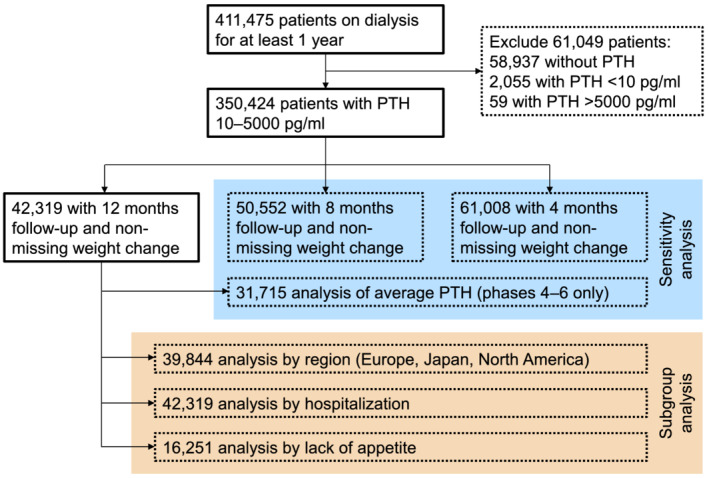
Study flow chart. PTH, parathyroid hormone.

The current analysis included 42 319 DOPPS participants from Phase 2 (2002–2004) through Phase 6 (2015–2018) in order to sample a wide distribution of PTH levels. Further details of the study sample are shown in *Figure* *1*. Country‐specific sample sizes in 12 month weight loss analysis are shown in Supporting Information, *Table* S1. Study patients had baseline PTH and post‐dialysis weight at least 1 year after haemodialysis initiation, and post‐dialysis weight measured 12 months later. PTH levels and weight changes commonly experienced by patients soon after initiating haemodialysis^24^ were not the subject of this study. Participants were recruited through 792 unique facilities from North America (the United States and Canada), Japan, Europe (Belgium, France, Germany, Italy, Spain, Sweden, and the United Kingdom), Australia, New Zealand, Russia, the Gulf Cooperation Council (Bahrain, Kuwait, Oman, Qatar, Saudi Arabia, and United Arab Emirates), Turkey, and China. Turkey and China were only included in the 12 month weight loss analysis, while the other countries were included in all analysis.

Individuals enrolled in multiple study phases were treated as independent observations and may be represented multiple times. Weight loss was defined as the 12 month weight minus the baseline weight. Patients with absolute or percent weight changes greater than the 99th percentile or less than the first percentile, within country (absolute weight change smaller than −20 to −9 kg or bigger than 6 to 14 kg; percent weight change smaller than −26% to −14% or bigger than 11% to 22%, depending on country), were excluded from all analyses.

For the majority of the study period, intact PTH assays were the only assay type available for clinical practice, and <10% of DOPPS facilities reported using bio‐intact (or whole) assays. PTH values from facilities reporting use of bio‐intact assays were multiplied by 1.7 to obtain an equivalent intact PTH value.^25^, ^26^ Missing or unknown assay types were assumed to be intact assays.

### Statistical analyses

Standard descriptive statistics were used to compare demographics and clinical characteristics of the study population according to baseline PTH levels. We used linear mixed models to estimate the association of baseline PTH levels with the percent weight change over 12 months. Random intercepts were included for each facility to account for potential clustering of outcomes by facility. We used multivariable models to incrementally adjust for potential confounding. Model 1 adjusted for country, study phase, and electronic health record data source (US Phases 4–6 only). Model 2 further adjusted for age, sex, time on dialysis, 13 comorbid conditions (as listed in *Table* *1*), single‐pool *Kt/V*, and dry weight. Model 3 further adjusted for albumin, haemoglobin, creatinine, calcium, and phosphorus. Model 4 further adjusted for calcium‐based binder, sevelamer, lanthanum, other phosphate binders, active vitamin D derivatives, and calcimimetics. We used Model 3 as the base model and Model 4 as a sensitivity analysis. Patients in the PTH category of 150–299 pg/mL were used as the analysis reference group. The percent weight change values were compared with the mean for this group when presenting results in figures. As the association between PTH and percent weight change may have differed geographically, we performed a stratified analysis by region (restricted to Europe, Japan, and North America). Similar stratified analyses based on body weight tertiles in each region, or based on lack of appetite were also performed. Additionally, because hospitalization may lead to marked weight loss, we separately examined the association between PTH and weight loss over 12 months among patients who were and were not hospitalized during this period.

**Table 1:** Baseline characteristics by PTH categories

Variable	PTH (pg/mL)					
	<50	50–149	150–299	300–449	450–599	≥600
	(*n* = 3169)	(*n* = 9035)	(*n* = 12 500)	(*n* = 7228)	(*n* = 4024)	(*n* = 6363)
Demographics						
Age, mean (SD), years	64.0 (13.4)	65.3 (13.5)	65.1 (13.7)	63.6 (14.4)	61.5 (14.6)	57.2 (15.3)
Male, %	57.4	58.6	59.0	57.5	56.4	54.6
Time on dialysis, mean (SD), years	6.6 (7.1)	5.5 (6.0)	4.9 (5.1)	5.0 (5.1)	5.3 (5.2)	5.9 (5.1)
Dry weight, mean (SD), kg	65.1 (18.8)	68.4 (19.8)	74.2 (21.1)	77.8 (21.9)	79.1 (22.4)	79.3 (23.3)
Single‐pool *Kt/V*, mean (SD)	1.50 (0.29)	1.52 (0.30)	1.55 (0.29)	1.56 (0.28)	1.56 (0.28)	1.55 (0.28)
Comorbidities, %						
Coronary artery disease	35.3	36.3	34.2	32.3	31.0	28.2
Congestive heart failure	25.1	25.7	27.1	24.8	27.4	25.0
Cerebrovascular disease	15.0	14.7	12.0	11.3	10.6	9.0
Peripheral vascular disease	21.3	22.7	22.5	22.1	21.2	18.0
Other cardiovascular disease	30.3	29.7	26.1	23.3	23.5	20.9
Hypertension	79.7	82.2	82.6	83.4	83.3	82.0
Diabetes	38.7	44.8	51.3	53.6	50.7	43.2
Neurologic disease	9.7	8.9	8.5	8.2	7.7	8.6
Psychiatric disease	13.1	14.4	16.5	17.9	19.7	19.6
Lung disease	8.2	9.7	9.8	9.3	9.5	8.3
Cancer	12.9	11.5	10.7	9.2	8.7	7.4
Gastrointestinal bleeding	4.5	4.3	4.2	4.5	4.6	3.9
Recurrent cellulitis	6.0	6.9	7.0	6.6	7.3	6.2
Laboratory tests						
Albumin, mean (SD), g/dL	3.7 (0.5)	3.7 (0.5)	3.8 (0.4)	3.8 (0.4)	3.8 (0.4)	3.8 (0.4)
Haemoglobin, mean (SD), g/dL	11.0 (1.4)	11.1 (1.4)	11.2 (1.3)	11.1 (1.3)	11.1 (1.4)	11.1 (1.4)
Creatinine, mean (SD), mg/dL	9.4 (3.0)	8.9 (3.0)	8.7 (2.9)	8.9 (2.9)	9.2 (2.9)	10.0 (3.1)
Calcium, mean (SD), mg/dL	9.2 (0.9)	9.1 (0.8)	9.0 (0.7)	9.0 (0.8)	9.0 (0.8)	9.0 (0.8)
Phosphorus, mean (SD), mg/dL	5.1 (1.6)	5.0 (1.5)	5.1 (1.5)	5.3 (1.5)	5.5 (1.6)	6.0 (1.8)
Total cholesterol, mean (SD), mg/dL	160 (39)	157 (40)	156 (42)	154 (41)	154 (40)	156 (41)
Medication, %						
ESA	88.5	88.3	87.4	86.6	86.5	86.8
Active vitamin D derivatives						
IV	12.8	24.3	39.8	49.5	51.9	48.5
Oral	35.4	29.9	26.7	26.6	28.4	26.6
Cinacalcet	6.0	11.0	13.4	17.3	21.4	32.3
Phosphate binder						
Calcium‐based	68.9	59.0	50.0	45.5	44.9	43.6
Lanthanum	7.2	9.4	7.7	7.3	7.4	7.2
Sevelamer	20.6	25.8	33.5	38.9	40.6	43.9
Other	6.6	7.3	7.8	9.3	10.2	12.2
Lack of appetite, %^a^						
Not at all	57.2	55.2	56.7	55.9	53.5	54.1
Somewhat	24.9	25.2	24.0	23.3	23.6	24.1
Moderately/very much/extremely	17.9	19.6	19.3	20.8	23.0	21.7

Abbreviations: ESA, erythropoiesis‐stimulating agent; IV, intravenous; PTH, parathyroid hormone.

^a^ Data on lack of appetite were available in 16 251 patients.

For mortality analysis, follow‐up time began at the end of the 12 month observation period of weight loss. That is, analysis included the period between baseline plus 12 months, until the first event of loss to follow‐up, transplantation, end of study phase, death, the most recent date of data availability within each study phase, or 7 days after leaving the facility due to transfer or change in kidney replacement therapy modality. To assess weight loss as a mediator between high PTH and mortality, the cut points for PTH and weight loss were chosen based on Cox models adjusting for covariates in Model 3 listed above, stratified by country, and accounting for facility clustering using robust sandwich covariance estimators. We then used accelerated failure time models with a generalized gamma‐distributed baseline to model patient survival (or relative life expectancy) from the baseline, in a manner similar to that described by VanderWeele.^27^ Errors in the coefficient estimates for model parameters and mediation effects were computed using a bootstrap approach (200 iterations).^28^ Total and direct effects of high PTH on mortality and the proportion mediated were reported. *Figure* S1 provides an illustration of the mediation analysis pathways between PTH, weight loss, and mortality (panel A), and the timing of the data used in the three major models for the mediation analysis (panel B).

As a sensitivity analysis, we estimated the association between average PTH levels during 4 months and the percent weight change during the subsequent 12 months: this analysis included only participants from Phases 4–6, because monthly laboratory values were not available in earlier phases. In addition, we based the analysis on patients with 4 and 8 month weight measurements after a baseline weight and PTH and estimated the association between baseline PTH and 4 or 8 month weight change. We also tested 4 or 8 month weight changes as mediators between PTH and mortality.

The proportion of missing data was <10% for all variables, except for single‐pool *Kt/V* (18%). We constructed a multiple‐imputation data set with 10 replicates using IVEware^29^; models were estimated separately for each replicate, and results were combined in standard fashion according to Rubin's rules.^30^ While it was used in the imputation process for other variables, lack of appetite was not imputed, due to concerns that some of the patients may have not answered the patient questionnaire because they were too sick to do so, and these patients may have non‐representative appetite scores even after adjustment. Patients with missing appetite information were thus excluded in the appetite subgroup analysis. All analyses were performed using SAS software, Version 9.4 (SAS Institute Inc).

## Results

### Patient characteristics

Among 42 319 patients at baseline, the mean (SD) body weight was 74 (22) kg, and the median PTH level was 251 pg/mL (interquartile range, 131–444 pg/mL, *Table* S2). Patients with higher PTH tended to be younger; have higher body weight, fewer comorbid conditions, and higher serum phosphorus levels; and be more often prescribed sevelamer, intravenous active vitamin D, and cinacalcet (*Table* *1*). Among patients with data on lack of appetite (*n* = 16 251), the degree of appetite loss was comparable across categories of PTH.

### Parathyroid hormone and weight change

**Figure 2:**
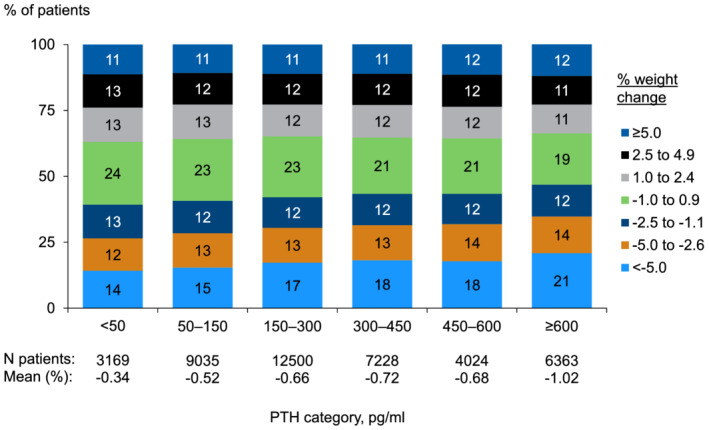
Distribution of 12 month percent weight change by baseline PTH level. PTH, parathyroid hormone.

Overall, the mean percent change in body weight for 12 months was −0.67%. Weight loss was more common than weight gain—11% of patients gained more than 5% weight, while 17% had more than 5% weight loss. Baseline PTH was inversely associated with 12 month weight change: mean 12 month weight change was −1.02%, −0.68%, −0.72%, −0.66%, −0.52%, and −0.34% for PTH ≥600, 450–600, 300–450, 50–150, and <50 pg/mL, respectively; the corresponding proportion of patients with 12 month weight loss >5% was 21%, 18%, 18%, 17%, 15%, and 14%, respectively (*Figure* *2*).

**Figure 3:**
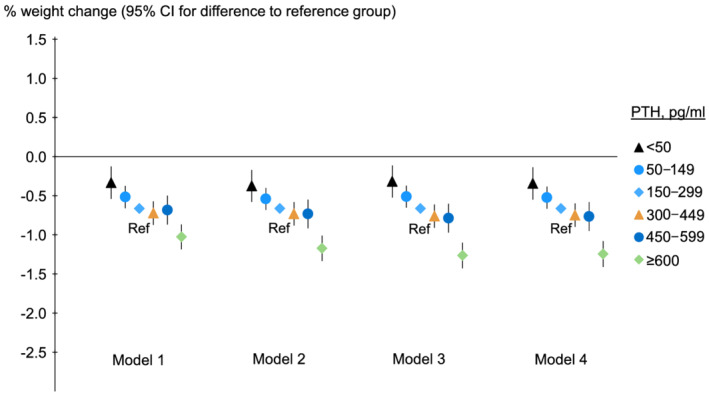
Association of baseline PTH with 12‐month percent weight change. PTH, parathyroid hormone; Ref, reference. Model 1 adjusted for country, study phase, and electronic health record data source (US Phases 4–6 only), accounting for facility clustering. Model 2 adjusted for covariates in Model 1 plus age, sex, time on dialysis, 13 comorbid conditions, single‐pool *Kt/V*, and dry weight. Model 3 adjusted for covariates in Model 2 plus albumin, haemoglobin, creatinine, calcium, and phosphorus. Model 4 adjusted for covariates in Model 3 plus calcium‐based binder, sevelamer, lanthanum, other phosphate binders, active vitamin D derivatives, and calcimimetics. The *P* value for trend was <0.001 for each model. The mean actual weight change was shown for the reference group, and other groups were plotted relative to the reference group based on adjusted model results.

Adjusted models also found a relationship between higher PTH and weight loss: 12 month weight change compared with PTH 150–299 pg/mL was −0.60%, −0.12%, −0.10%, +0.15%, and +0.34% for PTH ≥600, 450–600, 300–450, 50–150, and <50 pg/mL, respectively (*Figure* *3*, Model 3). The results were consistent across different models.

**Figure 4:**
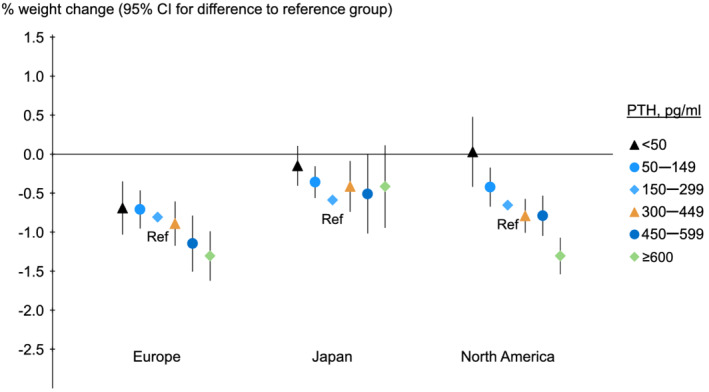
Association of baseline PTH with 12‐month percent weight change, by region. PTH, parathyroid hormone; Ref, reference. Model adjusted for country, study phase, electronic health record data source (US Phases 4–6 only), age, sex, time on dialysis, 13 comorbid conditions, single‐pool *Kt/V*, dry weight, albumin, haemoglobin, creatinine, calcium, and phosphorus, accounting for facility clustering. The *P* value for trend was <0.001 for Europe and north American, and 0.004 for Japan. The mean actual weight change was shown for the reference group, and other groups were plotted relative to the reference group based on adjusted model results.

The association between PTH and percent weight change was most dramatic in North America (*P* for interaction between PTH and region for outcome percent weight change, <0.001), where patients with PTH ≥600 pg/mL lost 0.65% [95% confidence interval (CI) −0.88%, −0.41%] more weight compared with those with PTH 150–299 pg/mL (*Figure* *4*). Patients with lower body weight had slightly larger percent weight changes, but direction of the associations between PTH and percent weight change were consistent across body weight tertiles in North America and Europe (*Figure* S2).

**Figure 5:**
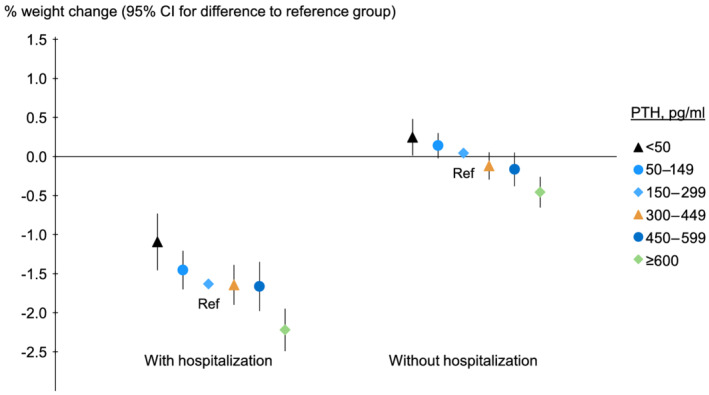
Association of baseline PTH with 12 month percent weight change in patients who were hospitalized and those who were not. PTH, parathyroid hormone; Ref, reference. Model adjusted for country, study phase, electronic health record data source (US Phases 4–6 only), age, sex, time on dialysis, 13 comorbid conditions, single‐pool *Kt/V*, dry weight, albumin, haemoglobin, creatinine, calcium, and phosphorus, accounting for facility clustering. The *P* value for trend was <0.001 for each analysis. The mean actual weight change was shown for the reference group, and other groups were plotted relative to the reference group based on adjusted model results.

**Figure 6:**
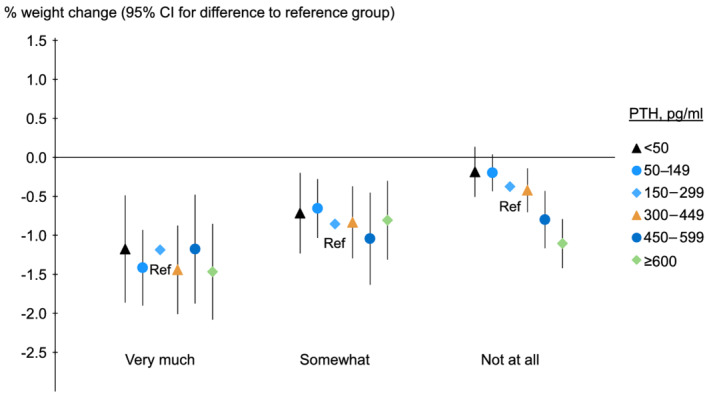
Association of baseline PTH with 12 month percent weight change, by lack of appetite. PTH, parathyroid hormone; Ref, reference. Model adjusted for country, study phase, electronic health record data source (US Phases 4–6 only), age, sex, time on dialysis, 13 comorbid conditions, single‐pool *Kt/V*, dry weight, albumin, haemoglobin, creatinine, calcium, and phosphorus, accounting for facility clustering. The analysis includes only individuals with available data on lack of appetite question (*n* = 16 251). The five possible responses to this question were as follows: not at all, somewhat, moderately, very much, and extremely. Categories of moderately, very much, and extremely were combined into a single category due to the small sample size. The *P* value for trend was 0.44 for very much, 0.11 for somewhat, and <0.001 for not at all. The mean actual weight change was shown for the reference group, and other groups were plotted relative to the reference group based on adjusted model results.

The mean percent weight loss was 1.56% for patients who were hospitalized during 12 months (*n* = 17 918) and 0.02% for those who were not hospitalized (*n* = 24 401). Regardless of whether patients were hospitalized or not, PTH was associated with percent weight change (*Figure* *5*). We also performed stratified analyses by lack of appetite in available samples. While patients with poor appetite showed larger percent weight changes, high PTH was associated with weight loss only in persons who endorsed preserved appetite (*Figure* *6*, *P* for interaction between PTH and lack of appetite for outcome percent weight change, 0.06).

### Assessment of weight loss as mediator between elevated parathyroid hormone and mortality

The association between percent weight change in 12 months and mortality suggests that the risk of mortality was higher for those with weight loss ≥2.5% than for any other group (*Figure* S3). For this reason, we used an indicator variable for weight loss ≥2.5% in the mediation analyses. For similar reasons (*Figure* S4), we used an indicator variable for PTH ≥600 pg/mL.

For analyses based on the weight change of 12 month measurement period, 14% (6125/42303) of patients died during median follow‐up of 1.0 years (interquartile range, 0.6–1.7 years). Among patients who survived at least 12 months after the PTH measurement, those with PTH ≥600 pg/mL had an 11% (95% CI, 8–12%) shorter lifespan, and 18% (95% CI, 14–23%) of this effect was mediated through weight loss ≥2.5%.

### Sensitivity analysis

**Figure 7:**
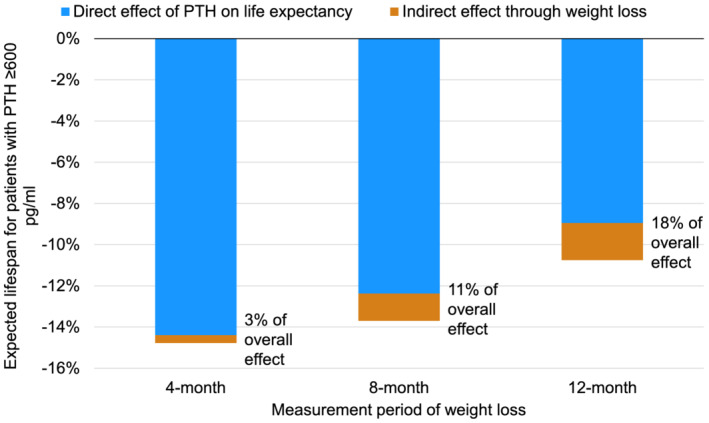
Association of baseline PTH ≥600 pg/mL with expected lifespan during post‐measurement follow‐up, mediated by weight loss ≥2.5% of body weight. PTH, parathyroid hormone. Model adjusted for country, study phase, electronic health record data source (US Phases 4–6 only), age, sex, time on dialysis, 13 comorbid conditions, single‐pool *Kt/V*, dry weight, albumin, haemoglobin, creatinine, calcium, and phosphorus, accounting for facility clustering.

When we used average PTH levels during 4 months as the exposure in participants from Phases 4–6 (*n* = 31 715), the association between PTH and percent weight change was qualitatively unchanged (*Figures* S5–S7). When we analysed percent weight change during 4 and 8 months (*n* = 61 008 and *n* = 50 552, respectively), the mean percent change in body weight for 4 and 8 months was −0.21% and −0.37%, respectively, compared with −0.67% for 12 months (*Figure* S8). In the mediation analysis, the indirect effect of PTH ≥600 pg/mL through the ≥2.5% weight loss over the 4 month period was 3% (95% CI 2–4%) of the 15% shorter lifespan during follow‐up. The indirect effect of PTH ≥600 pg/mL through the ≥2.5% weight loss over the 8‐month period was 11% (95% CI 9–13%) of the 14% shorter lifespan (*Figure* *7*).

## Discussion

In this international DOPPS cohort of over 42 000 haemodialysis patients, we found that elevated PTH was associated with subsequent weight loss, a hallmark of wasting. This relationship was sustained after adjustment for numerous covariates, was robust regardless of whether patients were hospitalized or not, and was more pronounced in persons with preserved appetite. Furthermore, the association between PTH and weight loss partly mediated the higher risk of mortality associated with elevated PTH levels. These findings suggest that SHPT may be a novel mechanism of wasting and that this pathway may be a mediator between elevated PTH levels and mortality among maintenance dialysis patients.

Wasting is a common feature of kidney failure. A recent meta‐analysis reported that approximately 28–54% of maintenance dialysis patients show evidence of wasting.^31^ Using data from the international DOPPS, we validate that weight loss, an important indicator of wasting, frequently occurs in this population: 31% of patients showed weight loss ≥2.5% during 12 months and 17% showed more than 5% weight loss. Such weight loss is a serious problem as it leads to poor clinical outcomes and increased mortality.^4^, ^5^ Our findings on the relationship between elevated PTH and weight loss support recent experimental evidence suggesting a direct role of PTH in adipose tissue browning^13^, ^14^ and indicate that targeting this pathway may hold promise for ameliorating wasting. Whether PTH‐lowering therapy can halt weight loss in haemodialysis patients with SHPT is important and worthy of further clinical research.

To our knowledge, this study is the first to show an association between elevated PTH and weight loss in a large cohort of haemodialysis patients. Our findings are consistent with a previous small study of haemodialysis patients showing that individuals with severe SHPT had increased resting energy expenditure.^19^ Similar findings have also been reported in patients with primary hyperparathyroidism^13^, ^14^ and cancer patients with detectable levels of PTH‐related protein, a tumour‐derived factor that shares the same receptor with PTH.^32^, ^33^ Our results extend these findings and further confirm that the association between PTH and weight loss persisted even after adjustment for potential confounders, an important approach that has not been systematically evaluated. The consistent findings across the diverse clinical settings support the key role of the PTH/PTH‐related protein pathway in the pathogenesis of wasting.

While we speculate that the strong association between PTH and weight loss is a clinical consequence of the direct effects of PTH on adipose tissue, other factors may have also been implicated. Because high PTH is associated with the risk of fracture^16^, ^34^, ^35^ and cardiovascular disease,^16^, ^17^ one may argue that the association between PTH and weight loss could be explained by such clinical events. However, the association persisted even when the analysis was restricted to patients who were not hospitalized. One could also postulate that the association between PTH and weight loss may simply reflect regression to the mean,^36^ given that patients with higher PTH tended to have higher body weight at baseline. However, this possibility is also unlikely because the association was sustained after adjustment for baseline body weight and was consistent across subgroups stratified by baseline body weight. Finally, it should be noted that we did not have a direct measure of fluid status, so we cannot exclude the possibility that the observed greater weight loss among persons with high baseline PTH levels resulted from greater reduction in extracellular fluid volume. However, the present analysis excluded those who started haemodialysis within 1 year, during which volume overload is actively corrected. Furthermore, patients with higher PTH levels have been shown to be more likely to miss haemodialysis treatments,^37^ suggesting poor patient adherence, which should rather lead to weight gain due to fluid retention through increased water and salt intake.^38^

Interestingly, we observed a stronger association between PTH and weight loss in North America. By contrast, the association was less pronounced in Japan, where there was almost no association between PTH levels ≥300 pg/mL and weight loss. These contrasting findings may be explained by the difference in the management of SHPT. A prior DOPPS analysis^17^ reported a steady increase over time in PTH levels in North America after the introduction of the international guideline suggesting a higher PTH target than previously recommended,^39^ while PTH levels remained stable in Japan where the local guideline recommends a much lower PTH target.^40^ Thus, patients with elevated PTH levels would be likely to receive pharmacotherapy to lower PTH levels or parathyroidectomy more quickly in Japan, which might attenuate the association between elevated baseline PTH and weight loss over subsequent months.

We also observed that the relationship between PTH and weight loss almost disappeared in patients with poor appetite. Loss of appetite is common in kidney failure patients and is associated with insufficient food intake, poor nutritional status, and inflammation.^6^, ^7^, ^8^ Importantly, inflammatory cytokines also contribute to adipose tissue browning and increased energy expenditure.^41^ As such, patients with poor appetite have multiple factors that lead to weight loss, obscuring the effect of PTH on weight loss that may occur through adipose tissue browning. Alternatively, there might be indirect mechanisms through which certain factors related to appetite loss attenuate the biological action of PTH on adipose tissue. Additional studies are needed to explore these possibilities.

Secondary hyperparathyroidism has been associated with increased risk of death.^16^, ^17^ In the Evaluation of Cinacalcet Hydrochloride Therapy to Lower Cardiovascular Events trial, cinacalcet did not significantly reduce the risk of death, but analyses adjusted for baseline covariates or accounting for study‐drug exposure showed a significant effect.^42^ However, the mechanisms underlying these observations have not been fully elucidated. One potential explanation is vascular calcification. However, PTH may not directly induce arterial calcification,^43^ and the associations between PTH and mortality have been independent of serum calcium and phosphorus,^16^, ^17^ potent inducers of calcification.^44^ Another possibility is fracture events, because high PTH levels are associated with fracture risk,^16^, ^34^, ^35^ and these events lead to a marked increase in subsequent mortality.^45^ However, the rates of fractures requiring hospitalization are approximately six‐fold lower than that of mortality in the DOPPS,^45^ indicating that fracture events could explain at most only a small fraction of the increased mortality with elevated PTH. In our study, we found by mediation analysis that weight loss accounted for 18% of the association between PTH and mortality. These findings highlight the possibility that weight loss is a clinically relevant pathway linking SHPT and mortality. It should be however noted that the analysis cannot infer causality, because the pathways of association were based on observational data and sensitive to assumptions inherent to mediation analysis.^27^ Future studies are needed to test whether weight loss contributes to the relationship between SHPT and mortality.

Our study has additional limitations. First, we lacked data on food intake. There is a possibility that food intake differs by PTH levels. However, elevated PTH remained to be associated with weight loss in an analysis restricted to individuals with preserved appetite. Furthermore, patients with higher PTH were younger and had higher body weight and fewer comorbidities, indicating a likelihood of higher food intake. If so, this should counteract the effects of PTH on weight loss. Of note, we did not use normalized protein catabolic rate as a surrogate of dietary protein intake, because it also reflects endogenous protein catabolism^46^ and thus could be on the causal pathway between SHPT and weight loss. Second, we evaluated weight loss for up to 12 months, which is a relatively short period. However, given the observed increasing magnitude of weight loss during the 12 month period and the progressive nature of SHPT,^15^ we expect that the association of SHPT with weight loss will be yet more pronounced over longer follow‐up. Third, we did not account for changes in PTH levels during longitudinal follow‐up. However, these levels remain relatively stable over time in the majority of the DOPPS participants,^47^ and we confirmed that the association between baseline PTH and subsequent weight loss was constant over time. Finally, multiple assays are available for measurement of PTH. Although the majority of DOPPS facilities reported using intact assays and we converted bio‐intact assay measurements into equivalent intact PTH values,^25^, ^26^ there may be large inter‐assay variability in PTH results^48^ and inter‐individual variability in the ratio of bio‐intact/intact PTH.^49^ Nonetheless, this variation would bias the association of PTH with weight loss toward the null. Strengths of our study include a large sample size, international data capture with a standardized protocol, prospective study design, and detailed covariate data that enabled us to control for potential confounders.

In conclusion, using data from the international DOPPS cohort, we showed that elevated PTH was associated with subsequent weight loss and this pathway partly mediated the association between elevated PTH levels and mortality in maintenance dialysis patients. These findings offer new evidence in support of a possible role of SHPT in the pathogenesis of wasting and weight loss. Future research should determine whether PTH‐lowering therapy can limit or prevent weight loss and improve longer‐term dialysis outcomes.

## Conflict of interest

H.K. has received honoraria, consulting fees, and/or grant support from Bayer Yakuhin, Chugai Pharmaceutical, Japan Tobacco, Kyowa Kirin, Novartis, and Ono Pharmaceutical. S.Y. has received honoraria from Kyowa Kirin. T.N. is an employee of Kyowa Kirin. P.E. has received honoraria, consulting fees, and/or grant support from Amgen, Sanofi, and Medice. J.Z., D.S.F., K.P.M., E.W.Y., and B.M.R. are employees for the non‐profit research organization Arbor Research Collaborative for Health, which has designed and carries out the DOPPS Program. B.M.R. has received consultancy fees or travel reimbursement from AstraZeneca, GlaxoSmithKline, and Kyowa Kirin, all paid directly to his institution of employment. M.F. has received honoraria, consulting fees, and/or grant support from Bayer Yakuhin, Fresenius Kabi, Kissei Pharmaceutical, Kyowa Kirin, Ono Pharmaceutical, and Torii Pharmaceutical. The remaining authors declare that they have no other relevant financial interests.

## Supporting information


**Table S1.** Number of patients included in 12‐month weight loss analysis by phase and country
**Table S2.** Distribution of baseline PTH by region
**Figure S1.** Illustration of mediation analysis. (A) Pathways between baseline PTH, weight loss, and mortality. (B) Timing of data used in three major models
**Figure S2.** Association of baseline PTH with 12‐month percent weight change, by body weight in Europe (A), Japan (B) and North America (C)
**Figure S3.** Association between percent weight change and mortality
**Figure S4.** Association between baseline PTH and mortality
**Figure S5.** Distribution of 12‐month percent weight change by baseline mean PTH level
**Figure S6.** Association of baseline mean PTH with 12‐month percent weight change
**Figure S7.** Association of baseline mean PTH with 12‐month percent weight change, by region
**Figure S8.** Association of baseline PTH with percent weight change in 4, 8, and 12 months
