# Supplementary material for: Secondary hyperparathyroidism, weight loss, and longer term mortality in haemodialysis patients: results from the DOPPS
Source: J Cachexia Sarcopenia Muscle. 2021 Jun 1;12(4):855–65. doi: 10.1002/jcsm.12722 (PMC8350219; doi:10.1002/jcsm.12722)
Supplement: Supplementary file 1 — Table S1. Number of patients included in 12‐month weight loss analysis by phase and country Table S2. Distribution of baseline PTH by region Figure S1. Illustration of mediation analysis. (A) Pathways between baseline PTH, weight loss, and mortality. (B) Timing of data used in three major models Figure S2. Association of baseline PTH with 12‐month percent weight change, by body weight in Europe (A), Japan (B) and North America (C) Figure S3. Association between percent weight change and mortality Figure S4. Association between baseline PTH and mortality Figure S5. Distribution of 12‐month percent weight change by baseline mean PTH level Figure S6. Association of baseline mean PTH with 12‐month percent weight change Figure S7. Association of baseline mean PTH with 12‐month percent weight change, by region Figure S8. Association of baseline PTH with percent weight change in 4, 8, and 12 months [file JCSM-12-855-s001.pdf]

Table S1. Number of patients included in 12-month weight loss analysis by phase and country.

	Phase 2 2002–2004	Phase 3 2005–2008	Phase 4 2009–2011	Phase 5 2012–2014	Phase 6 2015–2018	Total
ANZ	310	406	315	0	0	1,031
Belgium	292	295	351	270	131	1,339
Canada	358	341	298	409	353	1,759
China	0	0	0	563	0	563
France	237	395	354	90	0	1,076
GCC	0	0	0	383	254	637
Germany	300	471	476	513	239	1,999
Italy	318	394	462	385	130	1,689
Japan	1,173	1,494	1,474	1,540	1,600	7,281
Russia	0	0	0	189	0	189
Spain	373	409	515	354	339	1,990
Sweden	322	380	305	299	289	1,595
Turkey	0	0	0	55	0	55
UK	235	207	254	269	203	1,168
US	969	925	2,482	8,469	7,103	19,948
Total	4,887	5,717	7,286	13,788	10,641	42,319

Abbreviations: ANZ, Australia and New Zealand; GCC, Gulf Cooperation Council; UK, United Kingdom; US, United States.

Table S2. Distribution of baseline PTH by region.

	Europe (n = 10,856)	Japan (n = 7,281)	North America (n = 21,707)	Overall (n = 42,319)
PTH, pg/ml, median (IQR)	218 (109–390)	130 (68–223)	317 (192–522)	251 (131–444)
PTH ≥600 pg/ml, %	12.1	2.8	19.6	15.0

Abbreviations: IQR, interquartile range; PTH, parathyroid hormone.

Figure S1-A. Illustration of the mediation analysis pathways between baseline PTH, weight loss, and mortality.

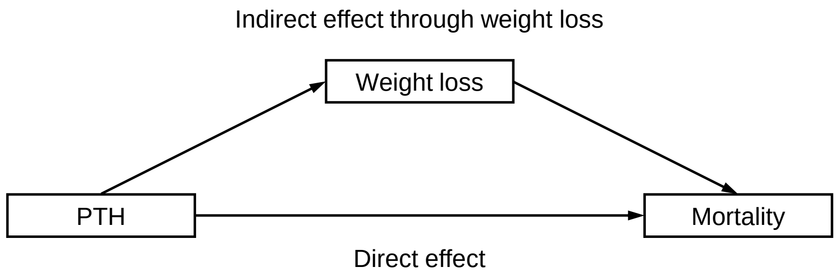

PTH, parathyroid hormone.

Figure S1-B. Illustration of the timing of the data used in the three major models for the mediation analysis.

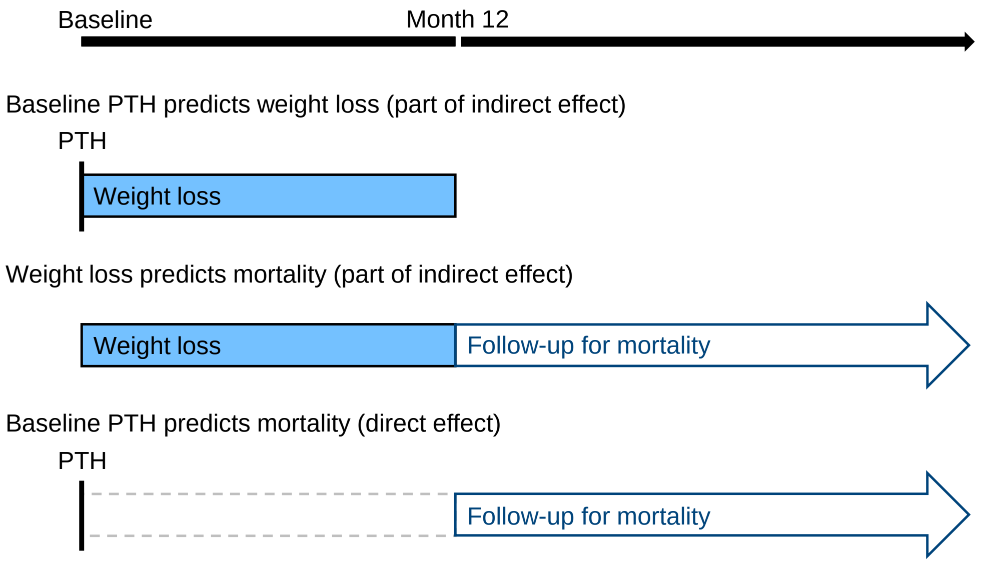

PTH, parathyroid hormone.

Figure S2-A. Association of baseline PTH with 12-month percent weight change, by body weight in Europe.

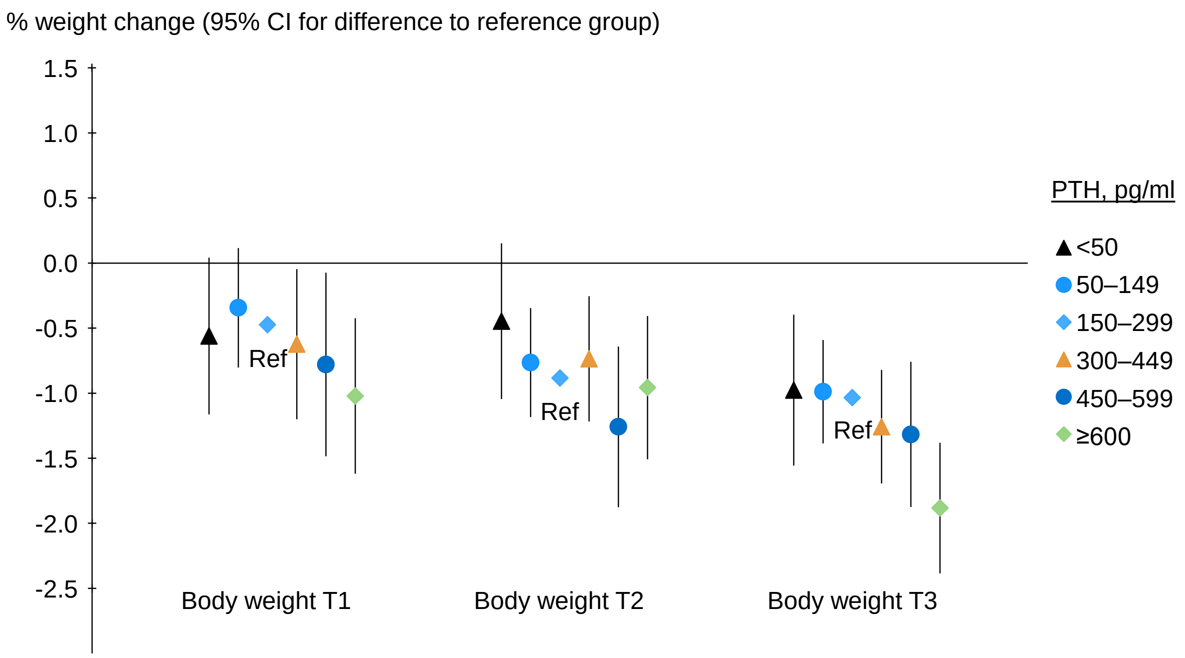

PTH, parathyroid hormone; Ref, reference. Model adjusted for country, study phase, electronic health record data source (US phases 4–6 only), age, sex, time on dialysis, 13 comorbid conditions, single-pool *Kt/V*, dry weight, albumin, hemoglobin, creatinine, calcium, and phosphorus, accounting for facility clustering. The *P* value for trend was 0.17 for tertile 1, 0.06 for tertile 2, and <0.001 for tertile 3. The mean actual weight change was shown for the reference group, and other groups were plotted relative to the reference group based on adjusted model results.

Figure S2-B. Association of baseline PTH with 12-month percent weight change, by body weight in Japan.

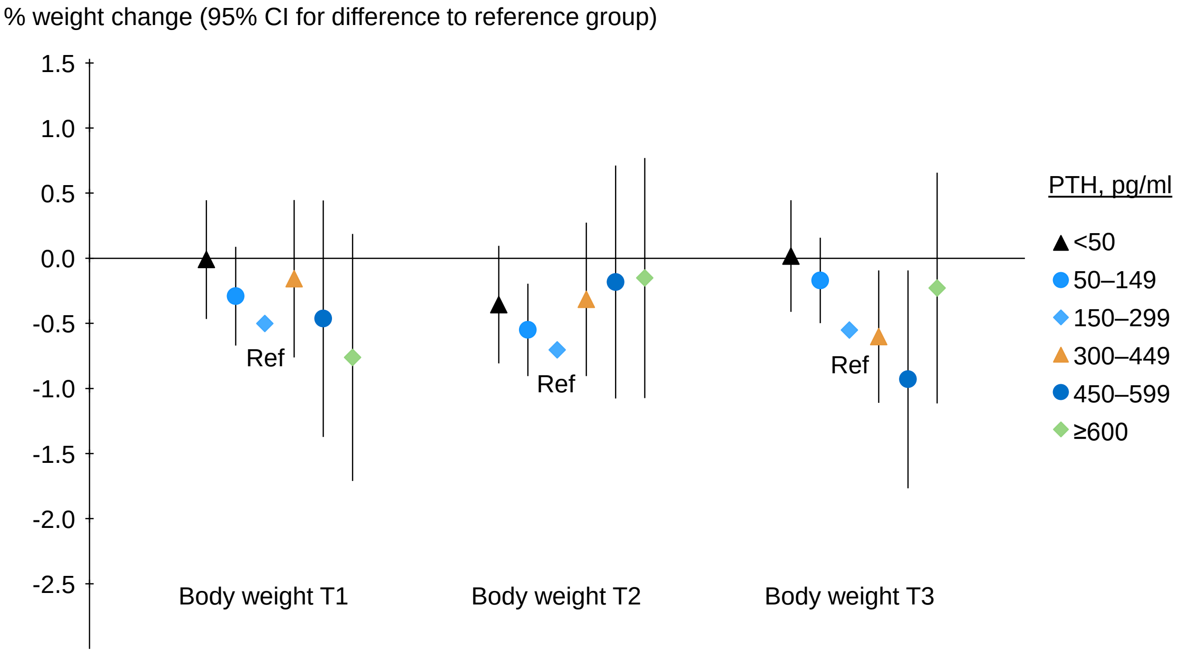

PTH, parathyroid hormone; Ref, reference. Model adjusted for country, study phase, electronic health record data source (US phases 4–6 only), age, sex, time on dialysis, 13 comorbid conditions, single-pool *Kt/V*, dry weight, albumin, hemoglobin, creatinine, calcium, and phosphorus, accounting for facility clustering. The *P* value for trend was 0.04 for tertile 1, 0.53 for tertile 2, and 0.009 for tertile 3. The mean actual weight change was shown for the reference group, and other groups were plotted relative to the reference group based on adjusted model results.

Figure S2-C. Association of baseline PTH with 12-month percent weight change, by body weight in North America.

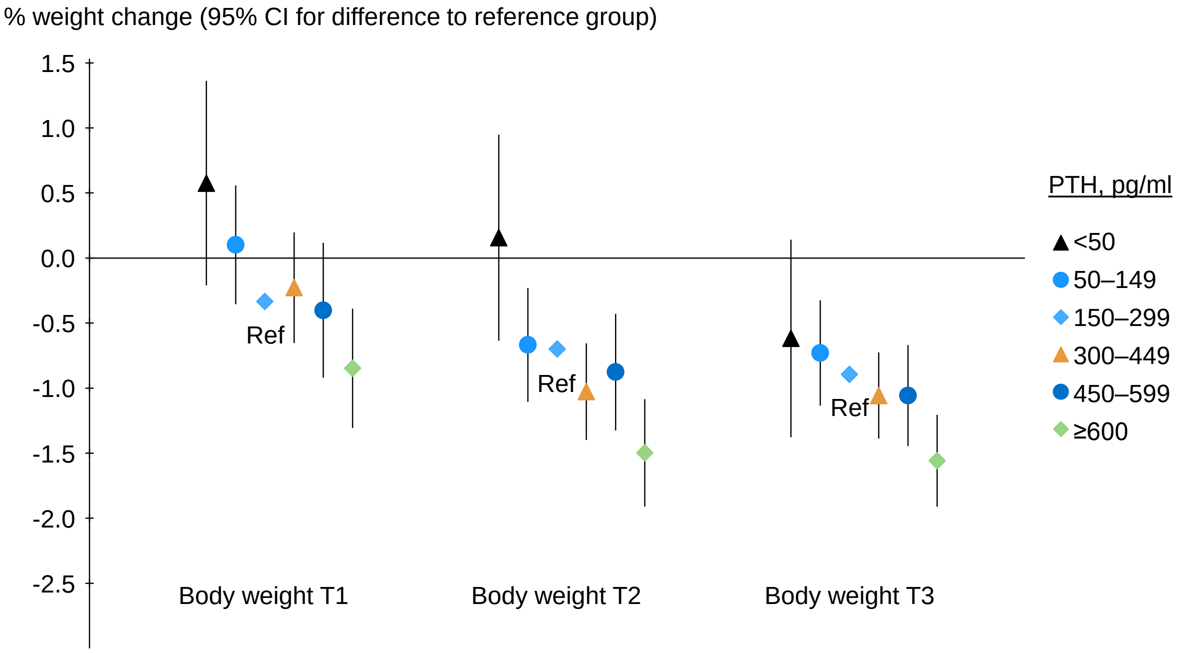

PTH, parathyroid hormone; Ref, reference. Model adjusted for country, study phase, electronic health record data source (US phases 4–6 only), age, sex, time on dialysis, 13 comorbid conditions, single-pool *Kt/V*, dry weight, albumin, hemoglobin, creatinine, calcium, and phosphorus, accounting for facility clustering. The *P* value for trend was <0.001 for each tertile. The mean actual weight change was shown for the reference group, and other groups were plotted relative to the reference group based on adjusted model results.

Figure S3. Association between percent weight change and mortality.

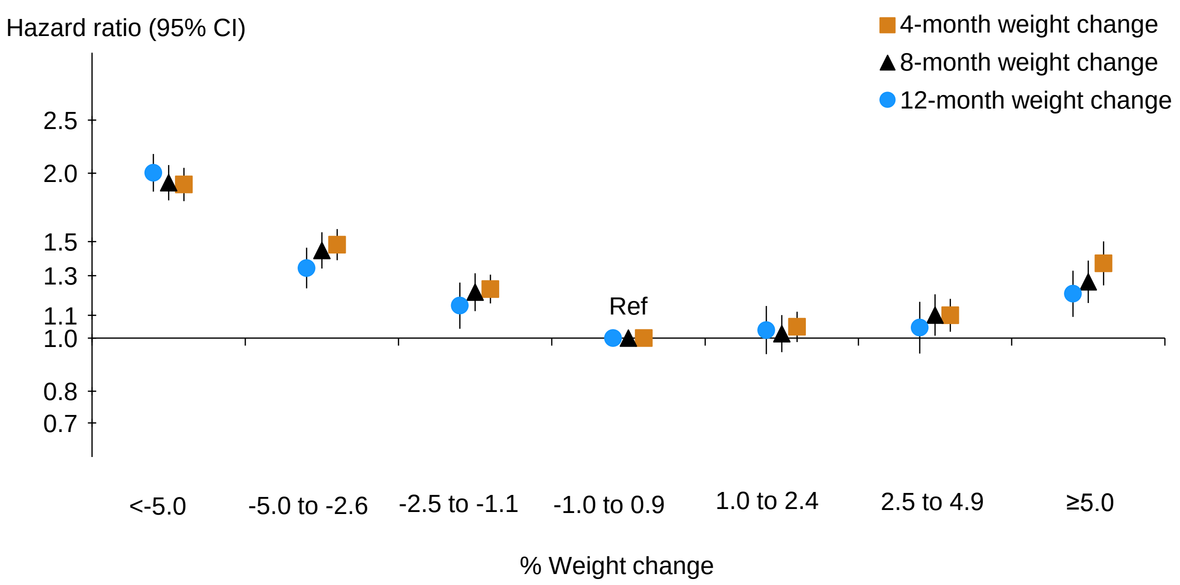

Model stratified by country, adjusted for study phase, electronic health record data source (US phases 4–6 only), age, sex, time on dialysis, 13 comorbid conditions, single-pool *Kt/V*, dry weight, albumin, hemoglobin, creatinine, calcium, and phosphorus, accounting for facility clustering.

Figure S4. Association between baseline PTH and mortality.

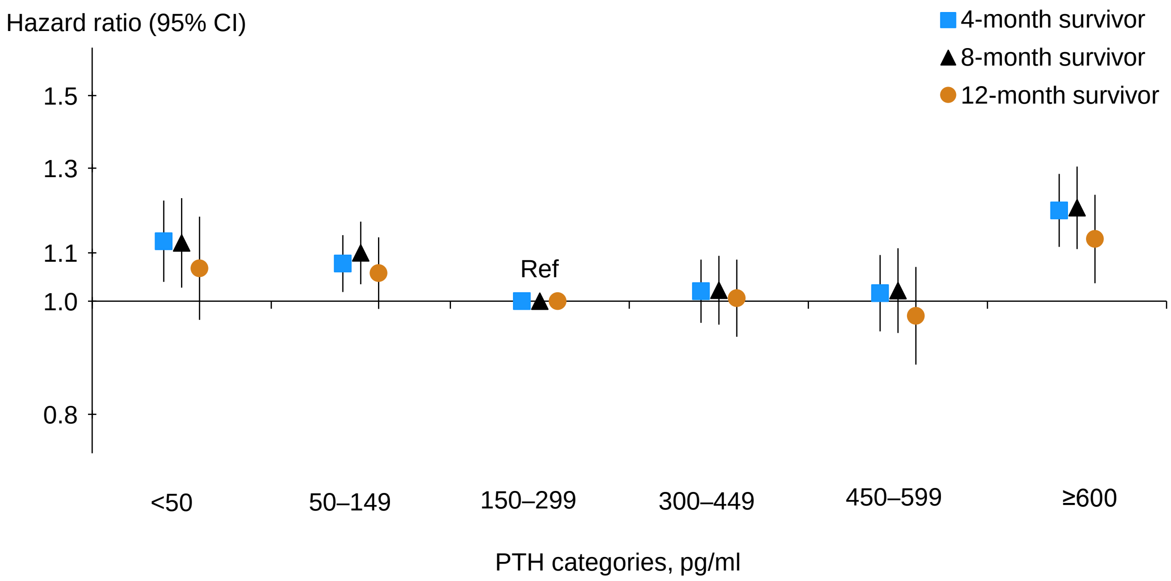

PTH, parathyroid hormone. Model stratified by country, adjusted for study phase, electronic health record data source (US phases 4–6 only), age, sex, time on dialysis, 13 comorbid conditions, single-pool *Kt/V*, dry weight, albumin, hemoglobin, creatinine, calcium, and phosphorus, accounting for facility clustering.

Figure S5. Distribution of 12-month percent weight change by baseline mean PTH level.

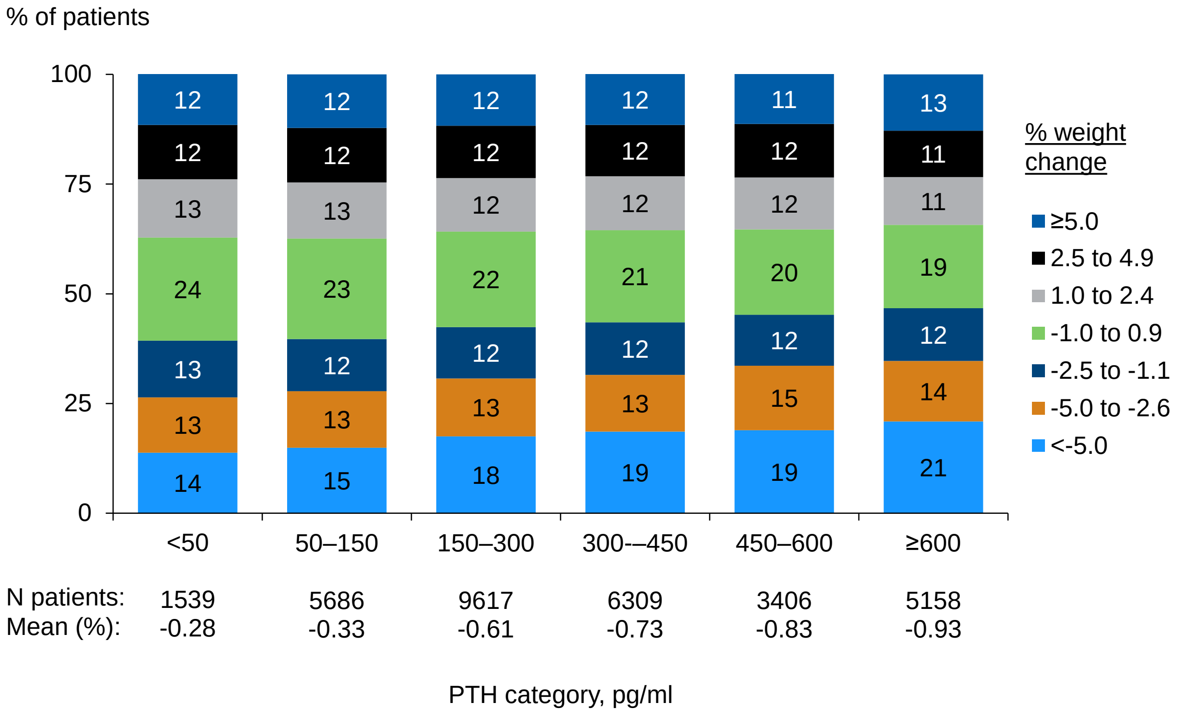

PTH, parathyroid hormone.

Figure S6. Association of baseline mean PTH with 12-month percent weight change.

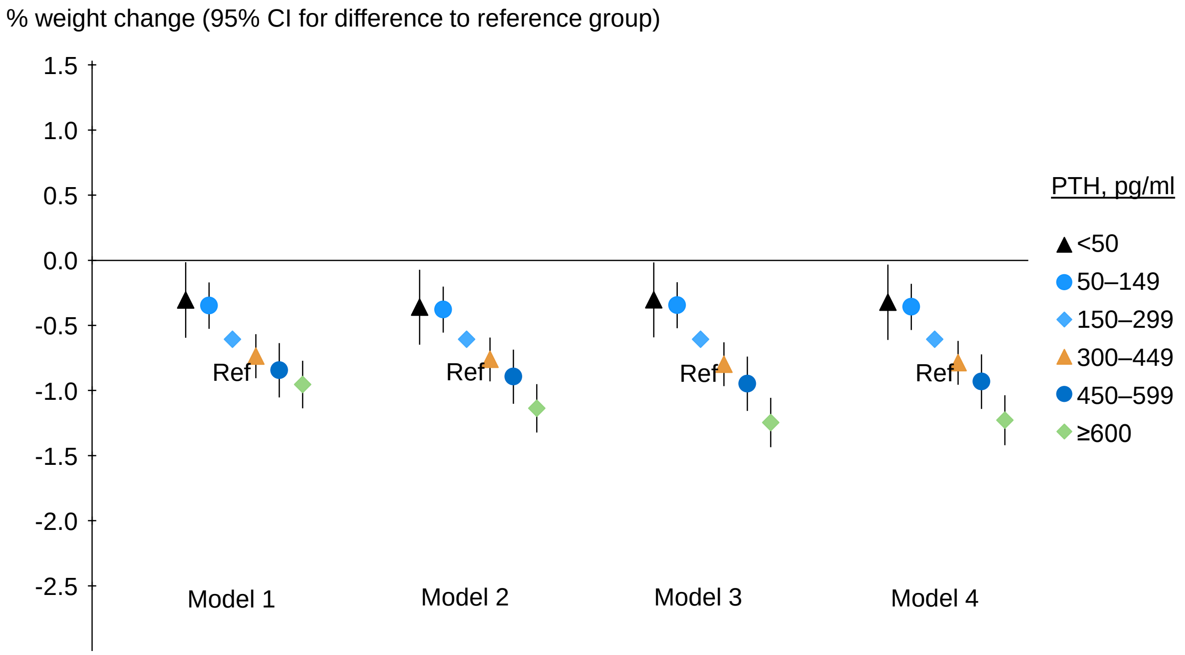

PTH, parathyroid hormone; Ref, reference. Model 1 adjusted for country, study phase, and electronic health record data source (US phases 4–6 only), accounting for facility clustering. Model 2 adjusted for covariates in Model 1 plus age, sex, time on dialysis, 13 comorbid conditions, single-pool *Kt/V*, and dry weight. Model 3 adjusted for covariates in Model 2 plus albumin, hemoglobin, creatinine, calcium, and phosphorus. Model 4 adjusted for covariates in Model 3 plus calcium-based binder, sevelamer, lanthanum, other phosphate binders, active vitamin D derivatives, and calcimimetics. The *P* value for trend was <0.001 for each model. The mean actual weight change was shown for the reference group, and other groups were plotted relative to the reference group based on adjusted model results.

Figure S7. Association of baseline mean PTH with 12-month percent weight change, by region.

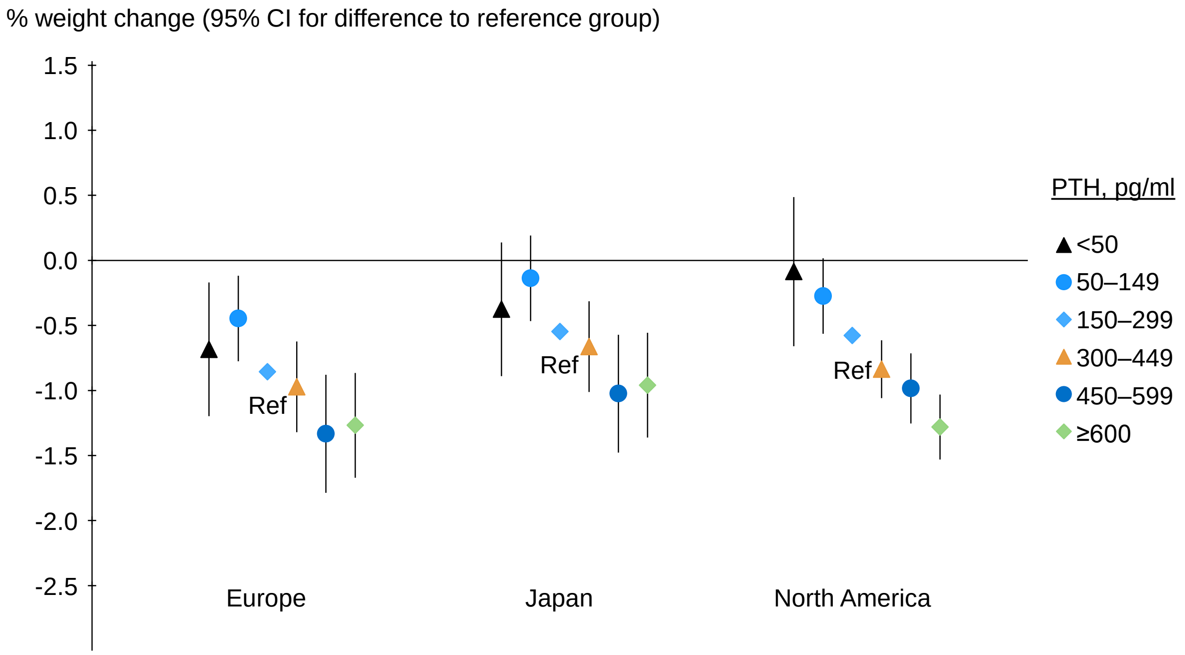

PTH, parathyroid hormone; Ref, reference. Model adjusted for country, study phase, electronic health record data source (US phases 4–6 only), age, sex, time on dialysis, 13 comorbid conditions, single-pool *Kt/V*, dry weight, albumin, hemoglobin, creatinine, calcium, and phosphorus, accounting for facility clustering. The *P* value for trend was <0.001 for Europe and North American, and 0.06 for Japan. The mean actual weight change was shown for the reference group, and other groups were plotted relative to the reference group based on adjusted model results.

Figure S8. Association of baseline PTH with percent weight change in 4, 8, and 12 months.

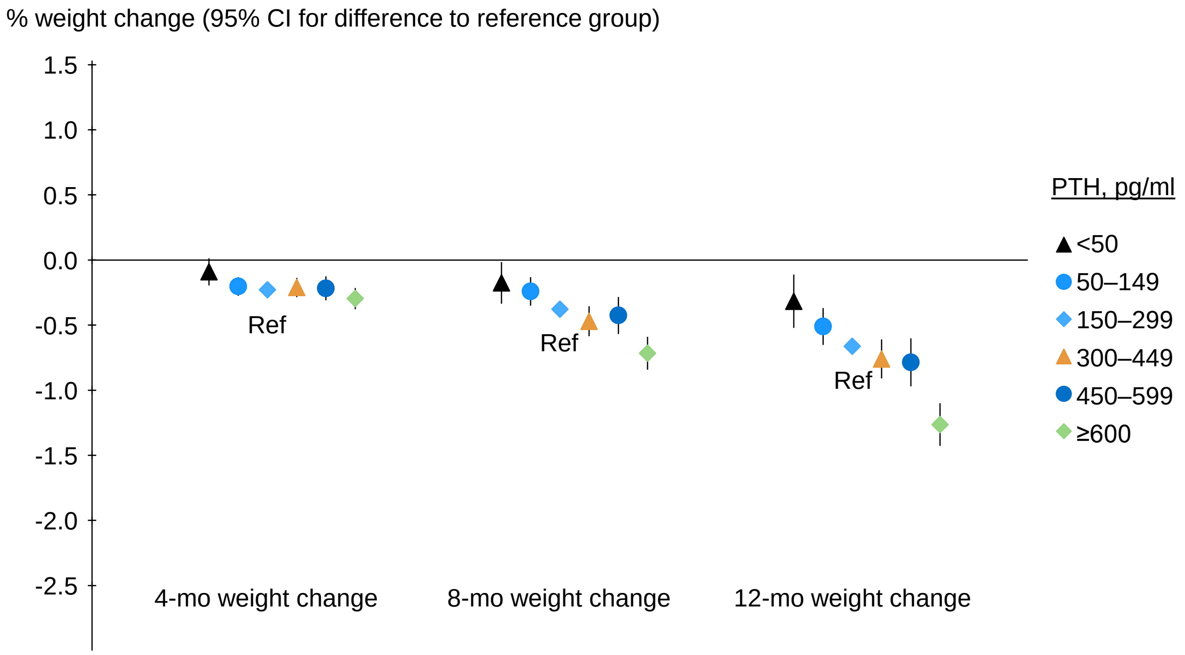

PTH, parathyroid hormone; Ref, reference. Model adjusted for country, study phase, electronic health record data source (US phases 4–6 only), age, sex, time on dialysis, 13 comorbid conditions, single-pool *Kt/V*, dry weight, albumin, hemoglobin, creatinine, calcium, and phosphorus, accounting for facility clustering. The *P* value for trend was <0.001 for each analysis. The mean actual weight change was shown for the reference group, and other groups were plotted relative to the reference group based on adjusted model results.
